# Hypervirulent Klebsiella pneumoniae causing aortitis retains its capsule and mucoviscosity and remains genotypically and phenotypically stable over time

**DOI:** 10.21203/rs.3.rs-6740913/v1

**Published:** 2025-08-05

**Authors:** Thomas A. Russo, Ulrike Carlino-MacDonald, Zachary J. Drayer, Connor J. Davies, Alan Hutson, Ting L. Luo, Melissa J. Martin, Patrick T. McGann, Francois Lebreton, Alan Sanders

**Affiliations:** University at Buffalo, State University of New York; University at Buffalo, State University of New York; University at Buffalo, State University of New York; University at Buffalo, State University of New York; Roswell Park Comprehensive Cancer Center; Walter Reed Army Institute of Research; Walter Reed Army Institute of Research; Walter Reed Army Institute of Research; Walter Reed Army Institute of Research; Albany Medical Center

## Abstract

Aortitis due to hypervirulent *Klebsiella pneumoniae* (hvKp) in a Belarusian male from central New York is described. Isolates at the time of diagnosis (Kp031824-1, Kp031824-2) and after 12 weeks of antimicrobial therapy (Kp070124) were characterized. Kp070124 was genomically and phenotypically unchanged retaining its capsular polysaccharide and mucoviscosity. *In vitro* studies established that capsule minus derivatives of Kp031824-1 and Kp031824-2 can occur due to mutations in *wcaJ*. But this genotype/phenotype was not selected for in Kp070124 after ≥15 weeks in the systemic compartment. Compared to capsule minus derivatives, the capsule positive phenotype demonstrated resistance to phagocytosis, but not to complement mediated bactericidal activity, suggesting resistance to phagocytosis is a more important defense mechanism at this site of infection. These data also support that a capsule positive, mucoviscous phenotype is selected for during infections in the systemic compartment. The surprising result that capsule positive strains have increased sensitivity to complement mediated bactericidal activity compared to capsule minus strains requires further investigation. The duration of therapy for this syndrome remains unclear but should be prolonged; adjunctive therapies (e.g. phage therapy, passive immunization, augmentation of cell mediated bactericidal activity) may be needed to overcome the protection endowed by the mucoviscous capsule of hvKp.

## Introduction

Hypervirulent *Klebsiella pneumoniae* (hvKp) is an emerging pathogen of increasing concern. The trademarks of hvKp infections are its ability to cause infections in the ambulatory setting with hepatic abscess and pneumonia being most common, multiple sites of infection, and usual sites of infection compared to classical *K. pneumoniae* such as endophthalmitis, central nervous system infections, and necrotizing fasciitis ^1^.

Although hvKp has been described to cause infection in nearly every site of the body, reports on vascular infection/mycotic aneurysm have been rare with cases primarily described from the Asian Pacific Rim ^2–5^. Further, in established and putative cases due to hvKp ^6–8^, genomic and phenotypic assessment of the offending strain, when performed, was limited to some combination of a string test, the identification of sequence type, capsule type, and 1–2 biomarkers. Given the rarity of this infection, optimal management, including the duration of antimicrobial therapy is uncertain, and in one case relapse occurred despite 5 weeks of antimicrobial therapy ^5^.

In addition, there are a few reports in which serial hvKp isolates have been obtained from patients over time and assessed ^9,10^. These data, combined with data from classical *K. pneumoniae* (cKp) have promoted the hypothesis that in the systemic compartment there is a positive selection pressure for the presence of capsule or increased capsule production, whereas in the urinary tract, selection pressure favors the loss of capsule ^11^. However, data is limited and inconsistent ^9,12,13^.

This report expands upon the existing knowledge base in three areas. First, we describe a case of aortitis due to hvKp for which the offending isolates underwent extensive genomic and phenotypic characterization. Second, a serial isolate from the aorta was recovered after 12 weeks of antimicrobial therapy and was also evaluated genomically and phenotypically. The aortic isolate was unchanged from the initial blood isolates despite a prolonged exposure to antimicrobial therapy; this suggests that for this systemic site either biofitness, which includes the presence of capsule, was optimized for survival or minimal replication occurred. Lastly the hvKp isolate was not eradicated despite a total of 12 weeks of antimicrobial therapy consistent with concerns that hvKp infections may require a longer treatment course than cKp. Taken together these data contribute to our understanding of the biology of hvKp infection and inform on management strategies needed for optimal outcomes.

## Results

### Clinical presentation and management of the aortitis

**Figure 1:**
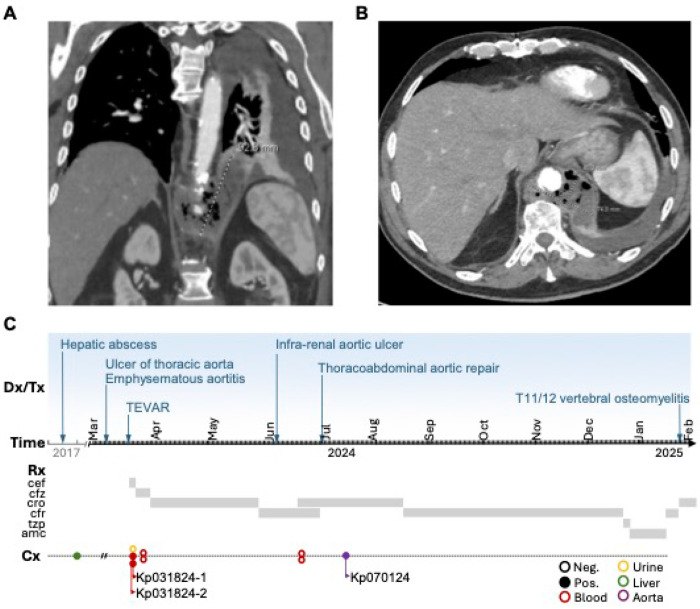
Clinical images and timeline. Panels a-b. Computerized tomography images demonstrating emphysematous aortitis. Panel c. Timeline of the clinical course delineating the timing of the infectious syndromes, surgical interventions, antimicrobial treatments, and culture results. TEVAR = endovascular graft of the thoracic aorta, cef = cefepime, cfz = cefazolin, cro = ceftriaxone, cfr = cefadroxil, tzp = piperacillin-tazobactam, amc = amoxicillin-clavulanate.

A 73-year-old Belarusian male with a past medical history of diabetes, diverticulosis, hepatic abscess in 2017, chronic leukopenia (2–3 ×10^9^/L), benign prostate hyperplasia, colonic polyps, coronary artery disease s/p myocardial infarction and CABG, and hypertension presented to Wynn Hospital (Utica, New York) in March 2024 with complaints of fatigue, fever, epigastric abdominal pain, dysphagia and occasional vomiting. He moved to central New York in 1989 and since has not travelled outside of the USA. A subdiaphragmatic ulcer of thoracic aorta and circumferential emphysematous thoracic aortitis was identified (Fig. 1A–B), which prompted transfer to Albany Medical Center (Albany New York) for further management. Upon admission, examination revealed a temperature of 102° F and vague abdominal pain without signs of peritonitis and laboratory findings demonstrated a WBC of 4.86×10^9^/L, diabetic ketoacidosis, and a creatinine of 5.15 mg/dL. A urine culture was unrevealing but *Klebsiella pneumoniae*, resistant only to ampicillin, was isolated from 2 independent blood cultures. Initial treatment with cefepime was de-escalated to cefazolin. No growth occurred with subsequent blood cultures. A CAT scan of the abdomen and pelvis confirmed aortitis but did not reveal any coincidental focus of inflammation or abscess.

An urgent endovascular graft of the thoracic aorta (TEVAR) was performed to stabilize the intimal ulcer and what appeared to be an emerging leak without aneurysmal defect. Ceftriaxone 2 grams IV q 12 hours was initiated for ongoing treatment of the emphysematous aortitis. A right axial femoral bypass was performed to offload the heart and maintain retrograde visceral organ perfusion in anticipation of an eventual open aortic resection and reconstructive surgery.

An 8-week course of ceftriaxone was completed as an outpatient followed by approximately a 4-week course of cefadroxil 500 mg PO BID. While still on cefadroxil, about three months after initial presentation, the patient returned to hospital with fever to 102° F and pain in the mid abdomen and back. C-reactive protein was 188 mg/L. Although a CAT scan of the abdomen performed two weeks earlier had demonstrated near resolution of the large emphysematous peri-aortic collection, repeat imaging demonstrated a new penetrating infra-renal aortic ulcer, and persistent inflammation of the aorta that contained the TEVAR graft. Blood cultures revealed no growth, and IV ceftriaxone 2 grams q 12 hours was restarted. Four days after admission, he underwent thoracoabdominal aortic repair with dacron tube graft, removal of the TEVAR graft, and a second infra renal aortic tube graft in the new area of ulceration. Other than a short post operative complication of spinal ischemia that resolved following lumbar drain decompression, the patient had an uneventful recovery. Notably, intra-operative culture of aortic tissue was positive for *Klebsiella pneumoniae* pathogen, with an identical sensitivity profile, as the blood culture isolates three months earlier.

The patient was discharged on hospital day 20 to complete eight weeks of IV ceftriaxone 2 grams q 12 hours. At the completion of this therapy, he was deemed to be in an excellent state of health and was started on an indefinite course of oral cefadroxil 500 mg BID. Three months later, a follow up CRP was 5.4 mg /L.

The patient was readmitted to Albany Medical Center in late December 2024 with acute sigmoid diverticulitis, without abscess, and treated medically with piperacillin-tazobactam for five (5) days followed by oral amoxicillin-clavulanate for 4 weeks; cefadroxil, was restarted in January 2025 after his treatment course with amoxicillin-clavulanate was completed.

However, in late January 2025, while on suppressive cefadroxil therapy, the patient represented with severe mid-back pain, and MRI imaging revealed discitis and vertebral osteomyelitis at the T11/12 region, which was dorsal to the Dacron aortic graft placed six (6) months earlier. Interventional radiology needle biopsy of the vertebral body yielded *Klebsiella pneumoniae* (isolate not available for study) with identical sensitivity pattern as all previous isolates. The patient was discharged on a 12-week course of IV ceftriaxone 2 g daily. The patient underwent an uneventful sigmoid resection in March 2025. Pathology revealed active diverticulitis with adhesions, and tissue culture grew vancomycin-resistant *Enterococcus faecium*, with no *Klebsiella* isolated. The patient was discharged from hospital to complete his 12-week course of IV ceftriaxone for his vertebral infection, with planned return to lifelong suppressive or cefadroxil therapy.

Interestingly, review of his remote medical records revealed that the hepatic abscess that was drained seven years prior to his emphysematous aortitis, yielded a *Klebsiella pneumoniae* with an identical sensitivity pattern (isolate unavailable). At that time the patient had evidence for coincidental sigmoid diverticulitis, CT scans also revealed significant chronic sigmoid diverticulosis raising the concern that this was a source for his hepatic infection. The patient was referred to colo-rectal surgery for consideration of a sigmoid resection, but surgery was not performed (see Fig. 1C for timeline of clinical events).

### The initial K. pneumoniae blood isolates possessed the hypervirulent genotype and phenotype

The two 3/18/2024 blood isolates Kp031824-1 and Kp031824-2 underwent whole genomic sequencing and analysis. Both isolates were genetically identical (0 SNPs apart), were sequence type (ST) 23, and possessed all 5 of the biomarkers present on the hvKp virulence plasmid that most strongly predict the hypervirulent phenotype (*iucA, iroB, peg-344, rmpA, rmpA2*) ^14^. Using the pLVPK sequence as a reference, the 3 isolates shared a large alignment fraction (> 94.9%; difference largely due to the lack of a single 5 kb region present in pLVPK that contained 9 CDS of mostly unknown function excepting one that was predicted as a putative TraD) and > 99.7% nucleotide identity, confirming the presence of the pLVPK-like plasmid (Supplementary Figure S1). The chromosomal KL1 capsule biosynthetic locus was identified as well as ICEKp10, which contained the biosynthetic genes for yersiniabactin and colibactin.

**Figure 2:**
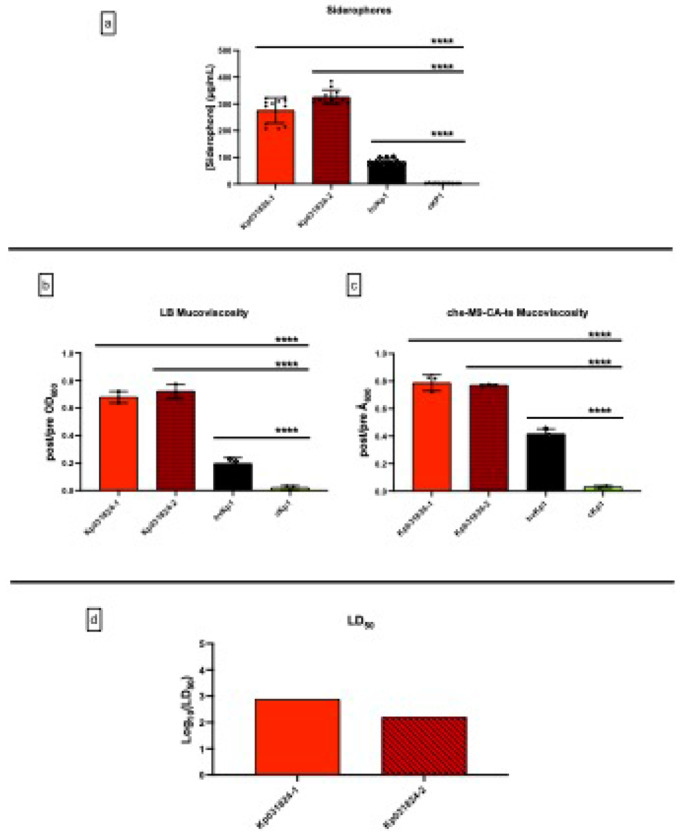
Comparison of *in vitro* quantitative siderophore production and mucoviscosity, and *in vivo* virulence between Kp031824-1 and Kp031824-2. Panel a. *In vitro* quantitative siderophore production for Kp031824-1 and Kp031824-2. Panels b-c. *In vitro* quantitative mucoviscosity for Kp031824-1 and Kp031824-2 grown in LB and che-m9-CA-te, respectively. A minimum of three biological replicates was performed for each strain for siderophore and mucoviscosity assays; hvKp1 and cKp1 were utilized as controls. Comparisons between Kp031824-1 and Kp031824-2 or hvKp1 and cKp1 were performed via ordinary one-way ANOVA, using Šidák’s multiple comparisons test (Prism 10.4.2 for MacIntosh, GraphPad Software Inc.). Panel d. Log_10_(LD_50_) values for Kp031824-1 and Kp031824-2. The LD_50_ was estimated using a logistic regression model with the factors for strain and inoculum (CFU/mL). A comparison between Kp031824-1 and Kp031824-2 was made by employing a blend of the empirical logit function along with least-squares regression incorporating strain and inoculum factors (CFU/mL) to derive p-values for comparing dose-response curves based on LS-means (SAS/STAT 15.1). All data except LD_50_ data are presented as the mean ± SD. ****p ≤ 0.0001, ***p ≤ 0.001, **p ≤ 0.01, *p ≤ 0.05.

**Figure 3:**
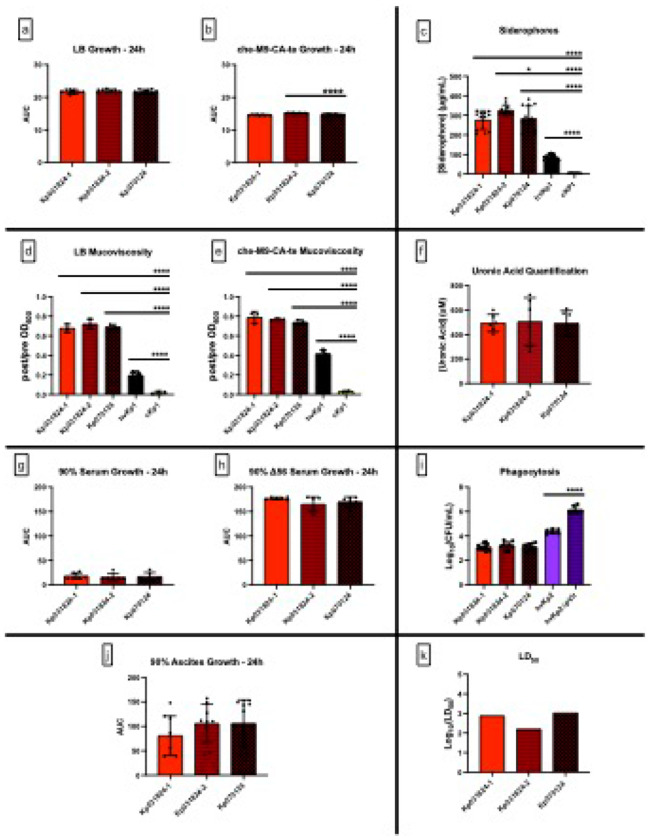
Comparisons of *in vitro* growth in LB, che-M9-CA-te, human serum, Δ56 serum, and ascites, *in vitro* quantitative siderophore production, mucoviscosity, uronic acid production, phagocytosis, and *in vivo* virulence between Kp031824-1, Kp031824-2, and Kp070124. Panels a-b. *In vitro* growth assessment of Kp031824-1, Kp0318234-2, and Kp070124 in LB and che-M9-CA-te, respectively. Growth was measured by OD_600_ over 24 hours, from which the area under the curve (AUC) was calculated. Panel c. *In vitro* quantitative siderophore production for Kp031824-1, Kp031824-2, and Kp070124. Panel d-e. *In vitro* quantitative mucoviscosity for Kp031824-1, Kp031824-2, and Kp070124 grown in LB and che-m9-CA-te, respectively. Panel f. In vitro uronic acid quantification for Kp031824-1, Kp031824-2, and Kp070124. Panels g-h. *In vitro* growth assessment of Kp031824-1, Kp031824-2, and Kp070124 in 90% human serum and Δ56 serum, respectively. Growth was measured via enumeration of colony-forming units (CFU) over 24 hours, from which the AUC was calculated. Panel i. *In vitro* quantitative assessment of Kp031824-1, Kp031824-2, and Kp070124 phagocytosis by J774A.1 murine macrophages. Data presented as Log_10_(CFU/mL), which is derived from the difference in concentration of surviving bacteria in cytochalasin D-treated wells vs untreated wells. Panel j. *In vitro* growth assessment of Kp031824-1, Kp0318234-2, and Kp070124 in 90% human ascites. Growth was measured via enumeration of CFU over 24h, from which the AUC was calculated. Panel k. Log_10_(LD_50_) values for Kp031824-1, Kp031824-2, and Kp070124. The LD_50_ was estimated using a logistic regression model with the factors for strain and inoculum (CFU/mL). Comparisons between strains were made by employing a blend of the empirical logit function along with least-squares regression incorporating strain and inoculum factors (CFU/mL) to derive p-values for comparing dose-response curves based on LS-means (SAS/STAT 15.1). A minimum of three biological replicates with three technical repeats was performed for each strain for all in vitro assays. hvKp1 and cKp1served as controls for siderophore and mucoviscosity assays, and hvKp2 and hvKp2ΔpVir were utilized as controls for phagocytic uptake. All data except LD_50_ data are presented as the mean ± SD. Apart from LD_50_ data, all comparisons between strains were made via ordinary one-way ANOVA, using Šidák’s multiple comparisons test (Prism 10.4.2 for MacIntosh, GraphPad Software Inc.). ****p ≤ 0.0001, ***p ≤ 0.001, **p ≤ 0.01, *p ≤ 0.05.

Next, quantitative siderophore production and mucoviscosity was assessed (Figs. 2a–c, Supplementary Table S1, S2). Kp031824-1 and Kp031824-2 produced significantly higher levels of siderophores in c-M9-CA (275.5 ± 47.28 μg/mL and 325.4 ± 26.22 μg/mL respectively) compared to the cKp control strain cKp1 (3.665 ± 0.650 μg/mL) (p < 0.0001). Likewise, Kp031824-1 and Kp031824-2 were significantly more mucoviscous in LB (post/pre OD_600_ 0.6788 ± 0.041 and 0.7221 ± 0.050 respectively) compared to the cKp control strain cKp1 (0.02 ± 0.016) (p < 0.0001) and in c-M9-CA-te (post/pre OD_600_ 0.789 ± 0.058 and 0.771 ± 0.008 respectively) compared to the cKp control strain cKp1 (0.031 ± 0.009) (p < 0.0001). Lastly, the LD_50_ for Kp031824-1 and Kp031824-2 for CD1 outbred mice that underwent SQ challenge were 7.63 x 10^2^ CFU and 1.59 x 10^2^ CFU respectively (Log_10_ values of 2.882 and 2.201 respectively) (Fig. 3d). Taken together genotypic and phenotypic data unequivocally demonstrate that Kp031824-1 and Kp031824-2 are hvKp isolates.

### The K. pneumoniae aortitis isolate after 12 weeks of antimicrobial therapy retained its capsular polysaccharide, mucoviscosity and the hypervirulent phenotype

Despite 8 weeks of ceftriaxone and approximately 4 weeks of cefadroxil, intraoperative cultures obtained during thoracoabdominal aortic repair on July 1st, 2024 resulted in the isolation of Kp070124. This isolate was present in this *in vivo* compartment for at least 106 days (the initial isolates were grown from blood obtained on March 18th, 2024). Genomic and phenotypic evaluation of this isolate would lend insight into *in vivo* selective pressures.

First, Kp070124 underwent whole genome sequencing and analysis, and these data were compared to the initial blood isolates Kp031824-1 and Kp031824-2. Surprisingly, no genomic changes were identified. Although this finding suggests that Kp070124, Kp031824-1, and Kp031824–2 should share an identical phenotype, transcriptional or post-transcriptional differences could not be excluded. Therefore, several phenotypic evaluations were performed.

First, growth and growth-related factors were assessed. The growth of Kp070124, Kp031824-1 and Kp031824-2 was similar in LB and numerically similar in c-M9-CA-te (Kp070124 versus Kp031824-2 was statistically but not biologically different) (Figs. 3a–b, Supplementary Table S1, S2). Further, Kp070124 produced similar levels of siderophores (283.1 ± 65.23 μg/mL) as Kp031824-1; Kp070124 and Kp031824-2 produced numerically similar levels that were statistically but not biologically different (Fig. 3c, Supplementary Table S1, S2). Next, bacterial defense factors/properties were assessed.

Kp070124 possessed similar mucoviscosity (post/pre OD_600_ 0.691 ± 0.024 and 0.740 ± 0.0230 respectively) as Kp031824-1 and Kp031824-2 in LB and c-M9-CA-te (Figs. 3d–e, Supplementary Table S1, S2). Capsule production for these strains, which was measured by quantitating uronic acid, was also similar (Fig. 3f, Supplementary Table S1, S2). Lastly, resistance to host-mediated bactericidal factors was determined. Kp070124 demonstrated a similar degree of resistance to both complement mediated bactericidal activity and macrophage mediated phagocytosis as Kp031824-1 and Kp031824-2 (Figs. 3g–h, 3i respectively). Lastly, an *ex vivo* and *in vivo* assessment was performed. The growth of Kp070124 was similar to Kp031824-1 and Kp031824-2 when grown *ex vivo* in human ascites (Fig. 3j, Supplementary Table S1, S2). Further, the LD_50_ for Kp070124 when CD1 outbred mice that underwent SQ challenge was 1.0 x 10^3^ CFU (Log_10_ value = 3.000), similar to that observed for Kp031824-1 and Kp031824-2 (Fig. 3k. Supplementary Table S1, S2).

These data demonstrate that Kp070124 has retained its hypervirulent phenotype: siderophore production, mucoviscosity, capsule production, resistance to complement mediated bactericidal activity and macrophage mediated phagocytosis, *ex vivo* growth in ascites, and lethality as measured by the LD_50_ in a murine systemic infection model were all stable despite prolonged *in vivo* selection pressures over at least 106 days.

### Despite antimicrobial pressure for approximately 12 weeks no evolution in antimicrobial resistance was observed.

It is not uncommon for antimicrobial resistance to evolve after a prolonged course of therapy. However, Kp070124 did not develop resistances nor were any genomic signatures predictive of resistance identified. The MIC for ceftriaxone against Kp031824-1, Kp031824-2, and Kp070124 as reported by the clinical microbiology laboratory was ≤ 1.0 μg/mL. To exclude small changes in resistance to ceftriaxone that may have conferred a selective advantage in the micro-environment of the infected aorta more expansive MIC testing was performed. However, no increase in the MIC for ceftriaxone against Kp070124 compared to Kp031824-1 and Kp031824-2 was observed (0.0625 μg/mL, 0.125 μg/mL, 0.125 μg/mL respectively).

### The initial K. pneumoniae isolates could evolve into capsule deficient derivatives

The observation that Kp070124 maintained a capsule supports that this phenotype is best suited for survival in this systemic compartment. However, this assumes that Kp031824-1 and Kp031824-2 could evolve a capsule minus phenotype. Therefore, Kp031824-1 and Kp031824-2 were assessed to determine the frequency of evolution to a capsule negative phenotype *in vitro*. Colonies of capsule positive bacteria appear yellow, whereas colonies of capsule negative bacteria appear grey. Therefore, 100–200 individual colonies of Kp031824-1 and Kp031824-2 were visually screened on LB agars plates after overnight growth in either LB or human ascites. After growth in LB, 2.1% and 3.1% of Kp031824-1 and Kp031824-2 colonies respectively appeared grey. After growth in ascites, 3.2% and 0% of Kp031824-1 and Kp031824-2 colonies respectively appeared grey. Conversely, grey derivatives appeared to be stable, reversions from a grey to yellow phenotype were not observed with serial passage.

**Figure 4:**
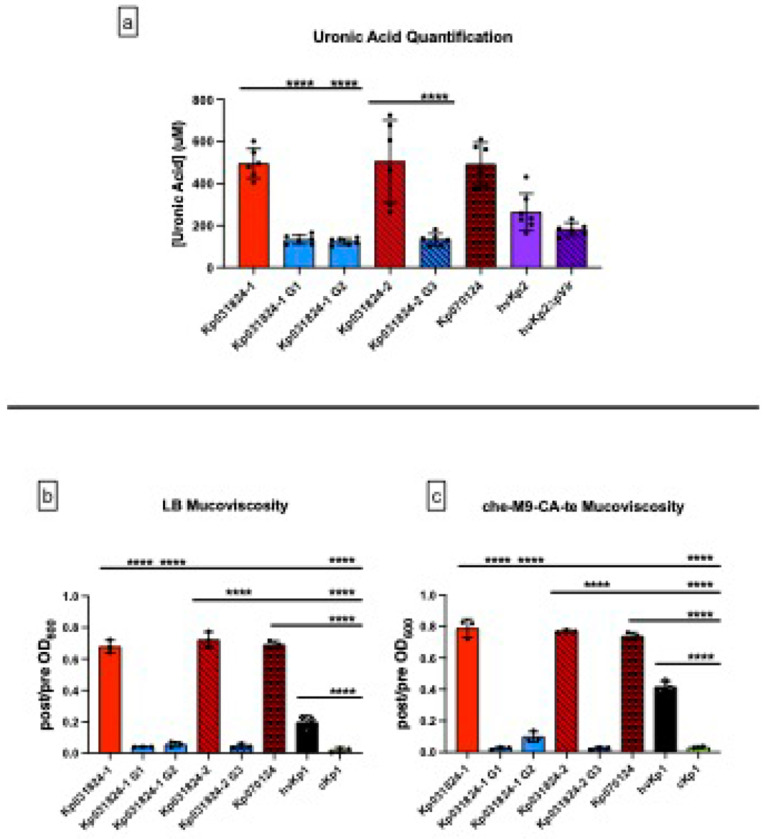
Comparison of *in vitro* quantitative uronic acid production and mucoviscosity between Kp031824-1, Kp031824-2, Kp070124, Kp031824-1 G1, Kp031824-1 G2, and Kp031824-2 G3 Panel a. *In vitro*uronic acid quantification for Kp031824-1, Kp031824-2, Kp070124, Kp031824-1 G1, Kp031824-1 G2, and Kp031824-2 G3. Panel b-c. *In vitro*quantitative mucoviscosity for Kp031824-1, Kp031824-2, Kp070124, Kp031824-1 G1, Kp031824-1 G2, and Kp031824-2 G3 grown in LB and che-m9-CA-te, respectively. A minimum of three biological replicates was performed for each strain. hvKp2 and hvKp2ΔpVir were utilized as controls for the uronic acid assay, hvKp1 and cKp1served as controls for siderophore and mucoviscosity assays. All data are presented as the mean ± SD. Comparisons between strains were made via ordinary one-way ANOVA, using Šidák’s multiple comparisons test (Prism 10.4.2 for MacIntosh, GraphPad Software Inc.). ****p ≤ 0.0001, ***p ≤ 0.001, **p ≤ 0.01, *p ≤ 0.05.

Three grey derivatives of Kp031824-1 and Kp031824-2, Kp031824-1 G1 (obtained after growth in LB), Kp031824-1 G2 (obtained after growth in ascites), and Kp031824-2 G3 (obtained after growth in LB), were assessed to confirm that this colonial morphology reflected the loss of capsule. First, Kp031824-1 G1, Kp031824-1 G2, and Kp031824-2 G3 underwent whole genomic sequencing to determine the basis for the observed phenotype using their wild-type parents are the reference strains. All three capsule minus derivatives were isogenic with their wild-type parents except for a mutation in *wcaJ* which encodes the glycosyltransferase WcaJ, a critical protein requisite for capsule synthesis ^15^; Kp031824-1 G1 had a stop codon at gln250, Kp031824-1 G2 had a val173glu substitution, and Kp031824-2 G3 had a Ile272 frameshift mutation leading to a premature stop codon at residue 287 (Supplementary Figure S2). To confirm that the identified mutations resulted in a significant decrease or loss of capsule, uronic acid was quantitated, which reflects capsule production. As expected, the mean uronic acid concentration was significantly less for Kp031824-1 G1, Kp031824-1 G2, and Kp031824-2 G3 (132.4 ± 21.82 μg/mL, 123.0 ± 15.43 μg/mL, 133.4 ± 28.85 μg/mL respectively) compared to their wild-type parents Kp031824-1 and Kp031824-2 (495.2 ± 70.95 μg/mL, 505.3 ± 195.5 μg/mL respectively, p < 0.0001) (Fig. 4a, Supplementary Table S1, S2). Mucoviscosity is also dependent on capsule production ^16^, and as expected, mucoviscosity was significantly decreased in Kp031824-1 G1, Kp031824-1 G2, and Kp031824-2 G3 compared to their wild-type parents after growth in both LB and c-M9-CA-te medium (p < 0.0001) (Fig. 4b–c, Supplementary Table S1, S2). These values are similar to what was observed for the cKp control strain cKp1 (Figs. 4b–c, Supplementary Table S1, S2). These data demonstrate that the grey colonial morphology reflects a significant decrease or loss of capsule and that Kp031824-1 and Kp031824-2 could evolve a capsule minus phenotype.

### Antimicrobial pressure does not appear to be a positive selection pressure for retention of capsule in vivo.

If Kp070124 was more resistant to ceftriaxone than the capsule-minus derivatives Kp031824-1 G1, Kp031824-1 G2, and Kp031824-2 G3 this would suggest the antimicrobial pressure could select for a capsule-positive phenotype. However, this was not the case. The MIC for ceftriaxone against Kp070124 compared to Kp031824-1 G1, Kp031824-1 G2, and Kp031824-2 G3 was similar (0.0625 μg/mL, 0.0625 μg/mL, 0.125 μg/mL, 0.125 μg/mL respectively).

### Resistance to phagocytosis but paradoxically not to complement mediated bactericidal activity appears to be a positive selection pressure for retention of capsule in vivo.

**Figure 5:**
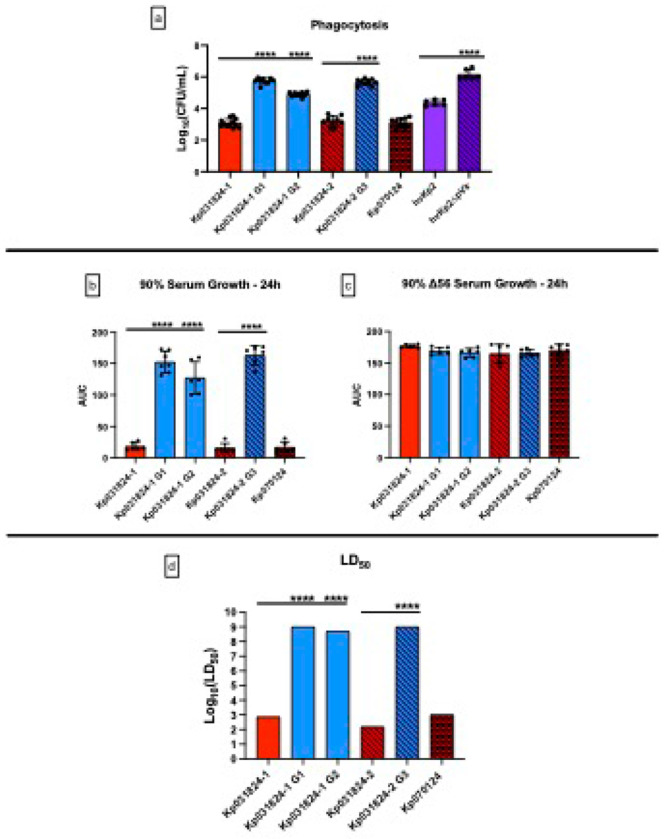
Comparison of *in vitro* quantitative phagocytosis, *in vitro* growth/survival in human serum and Δ56 serum, and *in vivo* assessment of virulence between Kp031824-1, Kp031824-2, Kp070124, Kp031824-1 G1, Kp031824-1 G2, and Kp031824-2 G3 Panel a. *In vitro* quantitative assessment of Kp031824-1, Kp031824-2, Kp070124, Kp031824-1 G1, Kp031824-1 G2, and Kp031824-2 G3 phagocytosis by J774A.1 murine macrophages. Data presented as Log_10_(CFU/mL), which is derived from the difference in concentration of surviving bacteria in cytochalasin D-treated wells vs untreated wells. Panel b-c. *In vitro* growth assessment of Kp031824-1, Kp031824-2, Kp070124, Kp031824-1 G1, Kp031824-1 G2, and Kp031824-2 G3 in 90% human serum and Δ56 serum, respectively. Growth was measured via enumeration of colony-forming units over 24 hours, from which the AUC was calculated. Panel d. Log_10_(LD_50_) values for Kp031824-1, Kp031824-2, Kp070124, Kp031824-1 G1, Kp031824-1 G2, and Kp031824-2 G3. The LD_50_ was estimated using a logistic regression model with the factors for strain and inoculum (CFU/mL). Comparisons between strains were made by employing a blend of the empirical logit function along with least-squares regression incorporating strain and inoculum factors (CFU/mL) to derive p-values for comparing dose-response curves based on LS-means (SAS/STAT 15.1). An upper cutoff value of Log_10_(LD_50_) equal to 9 was employed to correct for biologically implausible LD_50_ values. A minimum of three biological replicates with three technical repeats was performed for each strain for all in vitro assays. hvKp2 and hvKp2ΔpVir were utilized as controls for phagocytic uptake. All data excluding LD_50_ data are presented as the mean ± SD. Excluding LD_50_ data, all comparisons between strains were made via ordinary one-way ANOVA, using Šidák’s multiple comparisons test (Prism 10.4.2 for MacIntosh, GraphPad Software Inc.). ****p ≤ 0.0001, ***p ≤ 0.001, **p ≤ 0.01, *p ≤ 0.05.

Kp031824-1 and Kp031824-2 were compared to Kp031824-1 G1, Kp031824-1 G2, and Kp031824–2 G3 to generate mechanistic insights into which host defense factors mediated the positive selection for the retention of capsule within the systemic compartment. Not surprisingly the capsule positive wild-type parents Kp031824-1, Kp031824-2, and Kp070124 were significantly more resistant to phagocytosis than the capsule negative derivatives Kp031824-1 G1, Kp031824-1 G2, and Kp031824-2 G3 (Fig. 5a, p < 0.0001, Supplementary Table S1, S2). By contrast, Kp031824-1, Kp031824-2, and Kp070124 were significantly more sensitive to complement mediated bactericidal activity than Kp031824-1 G1, Kp031824-1 G2, and Kp031824-2 G3 (Figs. 5b–c, p < 0.0001, Supplementary Table S1, S2). Next, the LD_50_ for Kp031824-1 G1, Kp031824-1 G2, and Kp031824-2 G3 in CD1 outbred mice that underwent SQ challenge was measured. The LD_50_ for Kp031824-1 G1, Kp031824-1 G2, and Kp031824-2 G3 was > 7 (log_10_ CFU) (Fig. 5d, Supplementary Table S1, S2), a dose commensurate with the cKp phenotype. These data support that within the systemic compartment, selection for resistance to phagocytosis, which is mediated by the presence of capsular polysaccharide, appears to be more important than increased resistance to complement mediated bactericidal activity, which is mediated by the loss of capsular polysaccharide.

## Discussion

Vascular infection/mycotic aneurysm due to *K. pneumoniae* is uncommon. *Staphylococcus aureus*, Streptococci, and Salmonella are the most common offending pathogens ^17^. In this report, the most detailed genotypic and phenotypic assessment of a hvKp strain responsible for aortitis, including whole genomic sequence data, is described. It is unclear what proportion of *K. pneumoniae* mediated vascular infections/mycotic aneurysms is due to hvKp since most often the isolates have not been characterized. However, based on the clinical syndrome and/or strain characterization ^2–5^ and an incomplete but suggestive evaluation of the isolate ^6–8^ at least 4 cases due to hvKp and 3 cases putatively due to hvKp have been reported. From a clinical perspective, given the inability of the clinical microbiology laboratory to differentiate cKp from hvKp, it is best to assume that an hvKp isolate could be responsible for cases of vascular infection due to *K. pneumoniae* since this would affect management (e.g. vigilance for endophthalmitis, identifying occult abscesses) ^14^.

Several additional clinical caveats can be gleaned from this report. First, hvKp infections have been most described in the Asian Pacific Rim and in individuals of Asian, Pacific Islander, and Hispanic ethnic background ^1^. However, hvKp should not be excluded as the potential offending agent when these risk factors are absent as evidenced by this case. The infected Belarusian had resided in central New York since 1989 and had not travelled outside of the US. When and where this individual acquired the hvKp strain remains unclear, but there is some evidence that carriage of *K. pneumoniae* ST23/KL1 strains that possess all 5 of the biomarkers predictive of hvKp is occurring in health individuals ^18^. However, until hvKp strains are routinely identified in the clinical microbiology laboratory, clinical suspicion and consideration is paramount. In addition, despite 12 weeks of antimicrobial therapy, hvKp was still isolated from the site of infection. There is uncertainty as to whether hvKp infections require a more prolonged course of treatment compared to cKp. No prospective trial data is available, but several reports have described relapses of non-vascular infection ^19–22^, a mycotic aneurysm infection due to hvKp ^5^, and a mycotic aneurysm due to an uncharacterized *K. pneumoniae* strain ^23^. It is unclear if this is a unique characteristic of hvKp. However, biologically plausible mechanisms can be hypothesized that include the survival of the hvKp infecting strain at the site of initial infection due to its hypermucoviscous phenotype which could result in an *in vivo* biofilm even in the absence of foreign material or increased resistance to host factors such as professional phagocyte mediated bactericidal activity. Both scenarios could dictate the need for a more prolonged treatment course. In this case, one cannot exclude the possibility that failure was due to the presence of the endovascular graft. Lastly, it is intriguing that 7 years earlier the patient in this report had a liver abscess due to *K. pneumoniae*. That strain was unavailable for characterization, so it is unclear whether it was a hvKp isolate, and if so, whether it was the same as those in this report. However, if this isolate was a hvKp strain, it raises the question of whether prolonged colonization occurred and/or whether there is a genetic risk for infection.

Prior studies demonstrated that the loss or decreased production of capsule increases the ability to acquire exogenous DNA, including plasmids that assist in increased biofilm production, adherence to epithelial cells, and persistence in the murine urinary tract ^10,24^. But by contrast, the loss or decreased production of capsule decreases resistance to macrophage mediate phagocytosis and virulence in murine lethality infection models ^13,Song, 2024 #803,24^. These data support the hypothesis that genotypic/phenotypic changes such as the loss of or decreased capsule production that favors adhesion and biofim formation enhance colonization and persistence in certain niches where the host innate defense factors are less active ^25^. However, in systemic sites, such as the bloodstream, the presence of or increased capsule production protects against host-mediated bactericidal activity and increases survival. Data from this report are consistent with that hypothesis.

In this study, the capsule minus derivatives did not revert to the capsule positive phenotype after 6 passages. If there is not a program in which hvKp capsule minus strains can revert to a capsule positive phenotype then this could explain why colonization with hvKp does not necessarily result in infection; the capsule minus phenotype is needed for optimal adherence, which could facilitate invasion, but the capsule positive phenotype is needed for extraintestinal survival ^26^.

The loss or decreased production of capsule in *K. pneumoniae* resulted in decreased complement-mediated bactericidal activity in some ^12,24^, but not all studies ^10^. Our finding demonstrated that capsule deficient derivatives due to mutations in *wcaJ* were more resistant to complement-mediated bactericidal activity. Wang et al reported increased binding of the complement component C3b in similar capsule deficient derivatives ^24^. However, how this translates into increased complement resistance is unclear. By contrast, the loss of capsule resulted in increased susceptibility to macrophage phagocytosis. Since the capsule positive wild-type strain was selected for/retained *in vivo* in the human host in this study and was needed for the hypervirulent phenotype in a mouse systemic infection model, these data support the concept that resistance to phagocytosis is a more critical determinant than resistance to complement mediated bactericidal activity for at least the endovascular systemic compartment. It would be important to determine if a capsule positive phenotype is also selected for at other extraintestinal sites because if so, then adjunctive therapeutic interventions directed against capsules can be designed (e.g. bacteriophage that use capsule as their receptor or passive immunization directed against capsular antigens).

It was somewhat surprising that despite 12 weeks of antimicrobial pressure, resistance did not develop. We compared the initial blood isolates Kp031824-1 and Kp031824-2 to the subsequent aortic tissue isolate Kp070124 for changes in the ceftriaxone MIC below the susceptibility breakpoint (< 1 μg/mL), but no change was observed. Possible explanations include some combination of poor penetration of cephalosporins at the site of infection, biofilm formation, which may have been facilitated by the endovascular graft, functional resistance due to bacterial persisters ^27^, the development of small colony variants ^28^, or intracellular survival ^11^. But upon *in vitro* passage small colony variants were not observed. Further published data supports that at least *in vitro*, biofilm formation, cellular uptake and survival is facilitated by a capsule minus phenotype, contrasting the capsule positive of phenotype of Kp070124 ^9–11,13^. Therefore, it remains unclear why Kp070124 was able to persist within the human host despite ongoing antimicrobial treatment. However, if the increased capsule production and mucoviscosity inherent in hvKp strains is contributory, the use of rifampin as an adjunctive agent may be beneficial and warrants testing since it may decrease capsule production and mucoviscosity ^29^.

The biggest limitation of this study is that this is a case report with only a single serial human isolate available for evaluation. Nonetheless, the findings contribute to our understanding of the biology of hvKp infection and inform on potential management strategies that require further assessment. Further, whenever clinical isolates are evaluated, there is a concern that subsequent passage may have inadvertently affected the genotype/phenotype. However, this seems unlikely to have occurred for Kp031824-1, Kp031824-2, and Kp070124 since whole genomic sequencing did not identify any genomic changes between isolates, therefore lending confidence that genomic fidelity was retained.

In summary, until clinical microbiology laboratories are capable of accurately identifying hvKp strains, the clinician needs to maintain a high degree of suspicion, regardless of the geographic location and ethnic background of the patient, to guide management ^14^. Data that defines the risk of relapse/recurrence and the optimal duration of treatment for hvKp infections is needed. The determination of genotypic/phenotypic stability and the maintenance of capsule and mucoviscosty in hvKp serial isolates demonstrates the importance of this trait in the systemic compartment. Further, capsule promotes increased resistance to phagocytosis, but not complement mediated bactericidal activity compared to isogenic capsule minus derivatives. Taken together these data support that adjunctive therapies (e.g. phage therapy, antimicrobials, passive immunization, augmentation of cell mediated bactericidal activity) may be needed to overcome the protection endowed by mucoviscous capsular polysaccharide of hvKp.

## Methods

All methods were carried out in accordance with relevant guidelines and regulations. The procedures for obtaining human ascites fluid and serum were reviewed and approved by the Western New York Veterans Administration Institutional Review Board. Informed consent was obtained from all subjects donating blood for the isolation of serum. Informed consent for ascites fluid was waived because it was collected from de-identified patients who were undergoing therapeutic paracentesis for symptoms due to abdominal distension.

### Bacterial strains.

Kp031824-1 and Kp031824-2 were independent *K. pneumoniae* blood isolates from the case patient. Kp070124 was a tissue isolate obtained during the resection of the aortic aneurysm. hvKp1, hvKp2, hvKp87 (hvKp strains), and cKp1 (cKp strain) were variably used as controls for selected phenotypic assays and have been described ^30^. MRSN110821 was a *K. pneumoniae* isolate from Ukraine and possesses a cKp phenotype. Kp031824-1 G1 and Kp031824-2 G3 were spontaneous derivatives of Kp031824-1 and Kp031824-2 respectively that demonstrated a grey colonial morphology on agar plates after growth in lysogeny medium (5 g yeast extract, 10 g NaCl, 10 g tryptone, 15 g agar) (LB). This contrasts to the yellow colonial morphology of its progenitors Kp031824-1 and Kp031824-2. Kp031824-1 G2 was a spontaneous derivative of Kp031824-1 that demonstrated a grey colonial morphology on agar plates after growth in human ascites.

#### Whole genome sequencing, de novo assembly, annotations, and variant analysis.

All strains were sequenced using Illumina MiSeq short read platforms. Short read sequences were assembled *de novo* using Shovill (v1.0.9) with minimum assembly thresholds for contig size and coverage set at 200 bp and 49.5X, respectively. MLST assignment was performed using mlst v2.22.1 (https://github.com/tseemann/mlst). Comparisons of pLVPK plasmids were generated using the BLAST comparison tool version 1.4.1 integrated in Proksee.ca (PMID: 37140037). Parameters included: an expect value cutoff of 0.0001, filtering of low complexity regions, and filtering of regions with < 90% nucleotide identity. Single nucleotide polymorphism (SNP) calling was performed with Snippy v.4.4.5 (https://github.com/tseemann/snippy) using error corrected [Pilon v1.23] and annotated [Prokka v1.14.6] draft assemblies with isolate Kp031824-1 used as a reference. The core alignment length of the comparator isolates to the reference was 5.628 Mb. Snippy default parameters were used except for the minimum number of reads covering a site to be considered (--mincov - 20); and the minimum proportion of the reads which must differ from the reference (--minfrac - 80).

#### Quantitative siderophore assay.

Strains were grown overnight at 37°C in iron-chelated M9 minimal medium containing casamino acids (c-M9-CA) ^31^ and culture supernatants were assessed using the chromeazurol S dye assay as described ^32^. A minimum of 3 biological assays with 3 technical repeats were performed and the results were reported as the mean ± the SD.

#### Quantitative mucoviscosity assay.

Strains grown overnight in LB were used to inoculate 10 mL of either LB or c-M9-CA plus added trace elements (5 μg/mL CaCl_2_, 1 μg/mL CoCl_2_, 20 μg/mL MgCl_2_, 10 μg/mL MnCl_2_) (c-M9-CA-te) to a starting OD_600_ of approximately 0.2. Strains were grown at 37°C for 24 hours, and the assay was performed as described ^33^. A minimum of 3 biological assays were performed and the results were reported as the mean ± the SD.

## Growth assessment

Growth assays were performed in either LB, c-M9-CA-te, ascites, or serum as described ^34,36^. A minimum of 3 biological assays with 3 technical repeats were performed and the results were reported as the mean ± the SD.

### Quantitation of capsule.

Uronic acid (UA) content was measured as the means to quantitate capsule. The assay was performed largely as described ^35^. In brief, 2 mL of LB broth were inoculated for each strain and incubated overnight at 37°C. The following day, overnight cultures were diluted to an OD_600_ of 0.2, then incubated for 5 hours at 37°C; enumeration of these cultures established that titers were approximately 5 x 10^9^ CFU/mL. Five hundred uL of culture was combined with 100 uL 1% Zwittergent 3–08, heated at 50°C for 20 min, and centrifuged for 5 min at max speed. Three hundred uL of supernatant was combined with 1.2 mL absolute ethanol and incubated overnight at 4°C. The next day, the supernatant was decanted following 10 min centrifugation at 16,000 x g. The pellet was allowed to dry for 10 min, then resuspended in 200 uL diH_2_O, before being combined with 1.2 mL 12.5 mM tetraborate-sulfuric acid solution. After a 10 min incubation at 100°C, followed by 10 min on ice, 20 uL of 0.15% 3-phenylphenol was added and incubated at room temp for 5 min. One hundred uL of sample was plated in triplicate and the absorbance measured at 520 nm. Uronic acid concentration was determined via standard curve generated using glucuronolactone. A total of three biological assays with three technical replicates were performed for each strain.

## Phagocytosis assay

J774A.1 macrophages (ATCC TIB-67) were grown in Dulbecco’s modified Eagle’s medium (DMEM) containing L-glutamine, 4.5 g/L glucose, 10% heat-inactivated fetal bovine serum, and Gibco Pen Strep (10,000 units/L penicillin and 10,000 ug/L streptomycin). Upon achieving 90–95% confluency, the cells were washed and harvested by scraping in phosphate buffered saline (PBS). For use in phagocytosis assays cells were resuspended in growth medium lacking penicillin and streptomycin, seeded in 24-well plates at a density of 2.5 x 10^5^ cells/well, and allowed to reattach overnight at 37°C in 5% CO_2_. Bacterial cultures were grown in 2 mL of LB broth, shaking overnight at 37°C. The next day, bacterial overnight cultures were diluted to an OD_600_ of 0.05 in fresh LB and grown to mid-logarithmic phase (OD_600_ of approximately 0.4). Mid-logarithmic cultures were then centrifuged at 11,000 x g for 10 min, washed once with PBS, and diluted in DMEM to achieve a concentration of 5 x 10^6^ CFU/mL. Five hundred uL of diluted bacteria were added to each well with a resultant multiplicity of infection of 10 bacteria for every macrophage (calculated based on cell density seeded). The plates were then centrifuged at 170 x g for 5 minutes to increase contact between bacteria and host cells, and incubated for 30 minutes at 37°C.

Next, wells were washed three times with PBS and incubated with DMEM containing 300 ug/mL gentamicin for 15 min to eliminate extracellular bacteria. Following three washes with PBS, the macrophages were lysed with 0.1% Triton X-100 for 20 minutes and the lysate underwent serial 10-fold dilutions, which were plated on LB agar to enumerate bacteria. To confirm the bactericidal activity of gentamicin some wells, prior to infection, were incubated with cytochalasin D (2 μg/mL) for 30 minutes to inhibit bacterial uptake. The number of bacteria phagocytosed was the concentration of surviving bacteria from cytochalasin D-treated wells subtracted from the concentration of surviving bacteria from the corresponding untreated wells.

## CD1 mouse subcutaneous (SQ) challenge infection model / Ethics Statement

Animal studies were reviewed and approved by the Veterans Administration Institutional Animal Care Committee (IRBNet # 1580121) and the University at Buffalo-SUNY (N/A-Russo1) and were carried out in strict accordance with the recommendations in the guidelines delineated in the “NIH Guide for the Care and Use of Laboratory Animals”(revised 1985) and the “Ethics of Animal Experimentation Statement” (Canadian Council on Animal Care, July 1980) as monitored by the Institutional Animal Care and Use Committee (IACUC). All efforts were made to minimize suffering. Veterinary care for the animals was supplied by the staff of the Veterans Administration Animal Facility under the direction of a fully licensed veterinarian. Studies were performed as described ^30^. The various challenge inocula and number of mice used for each strain are delineated in Supplementary Table S3. Animals were closely monitored for 14 days after challenge for the study endpoints, which were survival or severe illness (*in extremis* state/death), which was recorded as a dichotomous variable. Signs that were monitored and which resulted in immediate euthanasia using methods consistent with the recommendations of the American Veterinary Medical Association Guidelines included hunched posture, ruffled fur, labored breathing, reluctance to move, photophobia, and dehydration.

### Minimal inhibitory concentration determination (MIC).

Bacterial strains were grown overnight in LB. Bacteria were diluted 1:1,000 in Mueller Hinton II cation adjusted broth (MH) and titers were confirmed by performing serial 10-fold dilutions and enumeration on LB plates. One hundred μL of diluted bacteria were added to a 96-well microtiter plate that contained various concentrations of ceftriaxone in 100 μL of MH with final concentrations ranging from 0.00195-32 ug/mL, resulting in a final bacterial concentration of approximately 5 x 10^5^ CFU/mL. Antimicrobial activity was determined by the measurement of optical density (OD_600_). Control wells contained medium only or bacteria without ceftriaxone. Plates were incubated a BioTek spectrophotometer for 20 hours, double-orbital shaking (237cpm (4mm), slow pace, 37°C) for 20 h at 37°C. Strains were assessed in parallel to control for inter-test variability. The MIC was defined as the lowest concentration of ceftriaxone that inhibited growth.

### Statistical Analyses.

*For in vitro* growth curves (Supplementary Figure S3) area under the curve was calculated as described ^36^. Desired comparisons between strains for experiments assessing siderophore production (Figs. 2a, 3c), mucoviscosity when grown in LB or c-M9-CA-te (Figs. 2b–c, 3d–e, 4b–c), uronic acid quantification (Figs. 3f, 4a), phagocytosis (Figs. 3i, 5a), and *in vitro* growth curves (Figs. 3a–b, 3g–h, 3j, 5b–c) were made via ordinary one-way ANOVA, using Šidák’s multiple comparisons test (Prism 10.4.2 for MacIntosh, GraphPad Software Inc.).

LD_50_ values were estimated using a logistic regression model as described ^37^. Pair-wise comparisons of the dose-response curves were used to generate LD_50_ values. Desired comparisons between LD_50_ values were made by employing a blend of the empirical logit function along with least-squares regression incorporating strain and inoculum factors (CFU/mL) to derive p-values for comparing dose-response curves based on LS-means (Figs. 2d, 3k, 5d).

## Supplementary Material

This is a list of supplementary files associated with this preprint. Click to download.

SupplementaryFigureS1.jpg

SupplementaryFigureS2.jpg

SupplementaryFigureS3.jpg

SupplementaryFigureS4.jpg

SupplementaryTable1.xlsx

SupplementaryTable2.xlsx

SupplementaryTable3.xlsx

SupplementaryFigurelegends.docx

## Data Availability

Both genomic assemblies and raw sequencing data of the isolates analysed in this study are publicly available in NCBI database under the Bio Project number PRJNA1265108
